# Diseases of the Eye

**Published:** 1892-07

**Authors:** 


Diseases of the Eye. A Hand-book of Ophthalmic Practice, for Students and Practitioners. By G. E. De Schweinitz, M. D., Professor of Diseases of the Eye in the Philadelphia Polyclinic; Lecturer on Medical Ophthalmoscopy in the University of Pennsylvania; Ophthalmic Surgeon to the Philadelphia Hospital, and to the Children's Hospital; Ophthalmologist to the Orthopædic Hospital and Infirmary for Nervous Diseases. With two hundred and sixteen illustrations and two chromo-lithographic plates. Philadelphia: W. B. Saunders, 913 Walnut Street. 1892. Price, cloth, $4.00 net; sheep, $5.00 net.

This book is divided into twenty-two chapters. The first chapter gives us the General Optical Principles, whilst chapter two instructs us in the Examination of the Patient and External Examination of the Eye. Chapter three speaks of Reflection; the Ophthalmoscope and its Theory; Ophthalmoscopy and Retinoscopy, whilst chapter four gives the student Normal and Abnormal Refraction. Chapters five to eleven treat of diseases of the eyelids; diseases of the Conjunctiva; diseases of the Cornea; diseases of the Sclera; diseases of the Iris; diseases of the Ciliary Body, and Sympathetic Irritation and Inflammation, and diseases of the Choroid. Chapter twelve gives all about Glaucoma. Chapters thirteen to sixteen the diseases of the Chrystalline Lens, Vitreous, Retina, and Optic Nerve. Chapters seventeen and eighteen treat of Amblyopia, Amaurosis, and Disturbances of Vision without Ophthalmoscopic changes; Amblyopia of the Visual Field, Scotomas, and Hemianopsia. Chapter nineteen, the Movements of the Eye-balls and their Anomalies. Chapters twenty and twenty-one, Diseases of the Lachrymal Apparatus and of the Orbit. Chapter twenty-two gives the Operations.

This is a new book and has been written in the hope that it may prove of service to students and practitioners who desire to begin the study of ophthalmology. We have spent several evenings in examining the book and are much pleased with it. To the neophyte it will be a great help, as the author has given the largest share of attention to the methods of examining the eyes, to the diagnosis and treatment of ocular diseases. The subject-matter has been given in greater detail than is customary in books written for students, and this is a step in the right direction. No physician, surgeon, or ophthalmologist will make a mistake in adding this beautiful work to his library.

The book contains some six hundred and forty pages of reading matter. The illustrations are first-class, and the paper, printing, and binding are all that can be desired. W. S.

